# Dietary intake is associated with the prevalence of uterine leiomyoma in Korean women: A retrospective cohort study

**DOI:** 10.1371/journal.pone.0291157

**Published:** 2024-02-15

**Authors:** Min-Jeong Kim, Sunmie Kim, Jin Ju Kim, Young Sun Kim, Ji Hyun Song, Jung Eun Lee, Jiyoung Youn, Sun Young Yang

**Affiliations:** 1 Department of Obstetrics and Gynecology, Division of Gynecology Oncology, CHA Hospital Ilsan Medical Center, Goyang-si, Gyonggi-do, Republic of Korea; 2 Department of Obstetrics and Gynecology, Seoul National University College of Medicine, Seoul National University Hospital Healthcare System Gangnam Center, Seoul, Republic of Korea; 3 Department of Internal Medicine and Healthcare Research Institute, Seoul National University Hospital, Healthcare System Gangnam Center, Seoul, Republic of Korea; 4 Department of Food and Nutrition, College of Human Ecology, Seoul National University, Seoul, Korea; 5 Research Institute of Human Ecology, Seoul National University, Seoul, Korea; University of Perugia: Universita degli Studi di Perugia, ITALY

## Abstract

**Objective:**

Uterine leiomyoma (UL), the most prevalent benign gynecologic tumor among reproductive-aged women, lacks sufficient research on the potential association between dietary intake and its occurrence in Korean women. Addressing this research gap, this study aims to evaluate the potential link between dietary intake and the prevalence of UL in Korean women.

**Methods:**

In this cross-sectional study, a cohort of 672 women, aged 23 to 73, were enrolled, with 383 (57%) being premenopausal. Dietary intake was assessed using a validated food frequency questionnaire (FFQ), and UL presence was determined through ultrasonography. The analysis focused exclusively on items within ten categories, including vegetables/fruit, vegetables, fruits, red meat, processed meat, poultry, fish, dairy product, milk, and alcohol. Multiple logistic regression models were employed to explore the relationship between dietary intake and the prevalence of UL, calculating odds ratios (ORs) and 95% confidence intervals (CIs) while adjusting for confounding factors.

**Results:**

Within the total cohort, 220 (32.7%) women were diagnosed with UL. High intakes of fish and poultry showed an association with higher UL prevalence. Odds ratios (95% CIs) for the upper quartiles compared to the lower quartiles were 1.68 (1.01–2.81; *p* trend = 0.05) for fish intake and 1.87 (1.11–3.17; *p* trend = 0.06) for poultry intake. Conversely, an inverse relationship emerged between dairy product intake and UL prevalence, with an odds ratio of 0.58 (95% CI 0.35–0.96; *p* trend = 0.05). Stratifying the analysis by menopausal status revealed a parallel pattern, with heightened UL prevalence with fish intake and reduced prevalence with dairy product intake. However, the link between poultry intake and UL prevalence was primarily observed among postmenopausal women. Among premenopausal women, elevated vegetable intake was linked to a decreased UL prevalence (OR 0.45, 95% CI 0.21–0.97 for top vs. bottom quartiles; *p* trend = 0.01).

**Conclusion:**

We found that high consumption of fish and poultry, coupled with low intake of dairy products, correlated with an elevated prevalence of UL. Furthermore, vegetable intake exhibited an inverse relationship with UL prevalence, particularly among premenopausal women.

## Introduction

Uterine leiomyoma (UL) is the most common benign gynecologic tumor, affecting approximately 25% of women of reproductive age, with peak prevalence occurring at age 50 and a lifetime risk of up to 70% [1–3]. While the pathophysiological mechanisms underlying the development of ULs at the cellular and molecular level have not been fully elucidated, they appear to be sex-hormone (estrogen and progesterone) dependent diseases, typically appearing after menarche, growing during reproductive ages, and regressing along with declining reproductive hormone levels after menopause [4–7]. Other known associated factors include age, ethnicity (with 2–3 times higher incidence in black women than in other races), genetics, number of pregnancies (more common in women who have had fewer pregnancies or deliveries), obesity, lack of physical exercise, and some dietary factors [8, 9].

As data on the relationship between dietary factors and malignant diseases such as breast or endometrial cancer, which are presumed to be estrogen dependent, have been reported mostly in terms of the potential of chemoprevention and long-term prognosis [10–13], the role of dietary nutrition as a factor that can be modified in the development and growth of UL has become a topic of interest, as dietary intake may alter either endocrine function or molecular biologic milieu [14].

According to previous studies, dietary patterns or some nutrients have shown significant associations with ULs. While the consumption of fruits and vegetables has shown a protective effect against ULs, findings have been inconsistent for other foods such as dairy, meat, or fish [8, 15–17]. Meanwhile, studies that have reported the association between nutritional intake analysis and the prevalence of UL in Korean women are limited. This study aimed to investigate the association between dietary intake and prevalence of UL stratified by menopausal status among Korean women who underwent both analysis of food intake and pelvic ultrasound exam from a previous cross-sectional study of our institute.

## Materials and methods

### Study design and participants

This study retrospectively used a prospectively collected cohort from our previous study [18]. Participants who underwent health checkups, including colonoscopy and dietary intake assessment, using a semi-quantitative food-frequency questionnaire (FFQ) at the Seoul National University Hospital Gangnam Center in Seoul, Korea, between May and December 2011, were registered. Among them, only women participants who also had pelvic ultrasonography during the study period were enrolled. Individuals who had already undergone hysterectomy or who did not take the pelvic ultrasound examination were excluded. This study was approved and has been granted an exemption from the requirement for additional consent procedures by the Institutional Review Board (IRB) of this institution. This exemption is based on the fact that the study involves the analysis of medical records from women who underwent pelvic ultrasound examinations among the participants of a previously conducted study at our institute [18]. To ensure the protection of personal information of the subjects included in this study, participants’ names were anonymized, and participant identification codes and medical record numbers were encrypted. Access to the data of the study participants was conducted for one year, from June 2015 to May 2016.

Postmenopausal status was defined as the absence of menstruation for at least 1 year. Women in peri-menopausal status (irregular cycles of more than ≥7 days differences or missed two or more cycles of menstruation within 12 months) were classified as premenopausal women [19].

### Clinical and laboratory assessment

Baseline characteristics, such as medication use (e.g., antidiabetic, antihypertensive, or lipid-lowering agents), underlying diseases (diabetes, hypertension, and dyslipidemia), smoking history, amount of physical activity, alcohol consumption, and reproductive characteristics (age at menarche, parity, age at first delivery, and menopausal status) were recorded during a medical interview using a structured questionnaire before a routine gynecologic examination. Anthropometric parameters (body mass index (BMI), waist circumference (WC), and blood pressure (BP)), and biochemical results (fasting plasma glucose, triglycerides, low-density lipoprotein (LDL)-cholesterol, and high-density lipoprotein (HDL)-cholesterol) were retrospectively reviewed for each individual, as previously described.

### Assessment of uterine leiomyoma

ULs were assessed through ultrasound examination using GE LOGIQ^®^9 (GE healthcare, General Electric Co., UK) equipment. The examination was performed by one of the three gynecologists who were obstetrics and gynecology specialists (M-J Kim, JJ Kim, and S Kim) with more than eight years of experience. The presence of UL was assessed only by intracavitary (mostly transvaginal, some transrectal) pelvic ultrasound examination, and cases with UL were defined as having one or more nodules of typical leiomyoma with ≥10mm in length.

### Assessment of dietary intake

Dietary intake data were assessed prior to the examination on the same day using a validated 106-item Food Frequency Questionnaire (FFQ) [20] with assistance from a registered dietician. Participants reported their usual frequency of consumption of various foods and typical portion sizes for the year preceding the interview date. Each food item had 9 options for frequency (ranging from “never or less than once per month” to “3 times per day”) and three options for portion size (‘small”, “medium”, or “large”). Fruit and vegetable intake included all raw, cooked, canned, frozen or dried forms of fruits and most edible vegetables. For the analysis, we examined the food consumption and total energy intake. Only items corresponding to the ten categories (vegetables/fruit, vegetables, fruits, red meat, processed meat (grouped into tertiles, two categories in postmenopausal women), poultry, fish, dairy product, milk, and alcohol (grouped into tertiles)) were included in the analyses and the amount of food intake was divided into quartiles. The median values of the tertiles or quartiles of each dietary group in all, pre-, and postmenopausal women are presented in S1 Table.

### Assessment of risk factors

Metabolic syndrome (MetS) was defined according to the harmonized definition proposed by the International Diabetes Federation/American Heart Association/National Heart, Lung, and Blood Institute [21]. A patient was diagnosed with MetS if they met three or more of the following criteria: abdominal obesity (waist circumference ≥85 cm for Korean women as proposed by the Korean Society for the Study of Obesity [22]), high triglycerides (TG) (≥150 mg/dL), low HDL-cholesterol (< 50 mg/dL), high fasting glucose(≥100 mg/dL) or treatment for diabetes, and increased blood pressure (≥130/85 mmHg) or treatment for hypertension.

Current smokers were defined as those who had been smoking at least one cigarette per day during the previous 12 months, while past-smokers were considered those who discontinued smoking for at least 12 months before inclusion in the study. “Ever smokers” refers to respondents who are current or past smokers.

Physical activity (PA) was measured by the modified Korean version of the PA questionnaire from the National Health and Nutrition Examination Survey [23]. PA was quantified using metabolic equivalent (MET)-minutes per week.

### Statistical analysis

Numerical variables were expressed as mean±standard deviation, and categorical variables were presented as numbers and percentages. If the parameters were not normally distributed, log_10_ transformation was used for analysis. The relationship between each dietary intake and UL was evaluated using binary logistic regression analyses. As ULs usually shrink after menopause due to a drastic drop in serum estrogen levels, the data were analyzed separately for two groups based on their menopausal status (premenopausal, including peri-menopausal, vs. postmenopausal). The median value of each tertile or quartile was included in the models as a continuous variable for trend testing. Odds ratios (ORs) and 95% confidence intervals (CIs) were calculated to evaluate the associations using multiple logistic regression models. In Model 1, we adjusted for confounding variables including age (years, continuous), BMI (kg/m^2^, <18.5, 18.5–23, 23–25, 25≤), total energy intake (kcal/d, quintile), and LDL-cholesterol (mg/dL, continuous). In Model 2, we further adjusted for all clinically relevant parameters, including menopausal status (premenopausal vs. postmenopausal), age at menarche (years old, ≤11, 11<), age at first delivery combined with parity (nulliparity, years old, <25, 25≤), alcohol intake (g/d, continuous), smoking status (never, or ever) and physical activity (MET-min/week, tertile). All analyses were performed using SAS 9.3 (SAS Institute Inc., Cary, NC, USA). We used 2-sided statistical tests, and *p*-values less than 0.05 were considered statistically significant.

## Results

A total of 672 women were included in the study, with an age range of 23–73 years (mean age 50.1 years), and 383 (57%) were premenopausal women. Among the entire study population, 220 individuals (32.7%) were diagnosed with UL, and there was no difference in prevalence of UL between pre- and postmenopausal women (34.7% vs. 30.1%, respectively; *p* = 0.21). Compared to women without UL, those with UL were older (51.0 ± 7.4 vs. 49.7 ± 9.5 years, *p* = 0.01), had a higher BMI (22.4 ± 3.2 vs. 21.9 ± 2.8 kg/ m^2^, *p* = 0.03), and higher LDL-cholesterol levels (128.2 ± 31.3 vs. 122.3 ± 32.5 mg/dL, *p* = 0.01). There were no significant differences in terms of reproductive, lifestyle, comorbidities, or laboratory parameters between the two groups (Table 1).

**Table 1:** Baseline characteristics of participants with and without uterine leiomyoma.

Variables	Women without UL (n = 452)	Women with UL (n = 220)	*p*-value
Age,years old	49.7 ± 9.5	51.0 ± 7.4	0.01
Size of UL, cm		2.3 ± 1.4	
Number of UL, n		1.8 ± 1.2	
Body mass index, kg/m^2^^†^	21.9 ± 2.8	22.4 ± 3.2	0.03
Abdominal circumference, cm^†^	80.0 ± 7.9	80.2 ± 9.6	0.73
SBP, mmHg^†^	111.2 ± 13.4	112.7 ± 12.4	0.14
DBP, mmHg^†^	69.8 ± 9.8	71.2 ± 9.6	0.10
Early menarche, n (%)^†^			0.20
Menarche at ≤11 years old	7 (1.6)	6 (3.2)	
Menarche at >11 years old	435 (98.4)	184 (96.8)	
Menopausal status			0.23
Premenopausal	250 (55.3)	133 (60.3)	
Postmenopausal	202 (44.7)	87 (39.7)	
Number of live births, n (%)			0.37
0	32 (7.1)	22 (10.0)	
1	51 (11.3)	28 (12.7)	
2	279 (61.7)	135 (61.4)	
≥3	90 (19.9)	35 (15.9)	
Age at first birth (years old), n (%)			0.15
<25	76 (19.1)	42 (24.4)	
≥25	321 (80.9)	130 (75.6)	
Smoking status, n (%)^†^			0.12
Never smoker	396 (90.8)	193 (92.3)	
Past smoker	18 (4.1)	12 (5.7)	
Current smoker	22 (5.0)	4 (1.9)	
Alcohol intake (g/d)^†^	6.1 ± 16.5	4.3 ± 10.3	0.70
Physical activity (MET-minute/week)	1140.1 ± 2569.4	1037.3 ± 1905.9	>0.99
Hypertension, n (%)^†^	83 (19.1)	50 (23.5)	0.20
Diabetes mellitus, n (%)^†^	68 (15.9)	31 (14.7)	0.69
Dyslipidemia, n (%)^†^	138 (31.9)	57 (26.9)	0.20
Metabolic syndrome, n (%)^†^	52 (12.2)	21 (9.9)	0.39
Glucose, g/dL^†^	89.9 ± 15.7	90.4 ± 14.4	0.56
Triglyceride, mg/dL^†^	75.6 ± 42.9	74.8 ± 38.0	0.74
HDL-cholesterol,mg/dL^†^	57.0 ± 10.9	57.4 ± 10.6	0.58
LDL-cholesterol, mg/dL^†^	122.3 ± 32.5	128.2 ± 31.3	0.01
25OH-D_,_ ng/mL^†^	22.2 ± 7.3	22.2 ± 7.5	0.80

*Abbreviations; UL, uterine leiomyoma; SBP, systolic blood pressure; DBP, diastolic blood pressure; HDL, high-density lipoprotein; LDL, low density lipoprotein; MET, metabolic equivalent; 25OH-D, 25-hydroxyvitamin D

†The total number of participants was different because of missing values.

Data are shown as mean ± standard deviation or numbers (percentages)

The mean number of UL nodules and dimensions of the largest myomas are presented in Table 1. The largest diameter was larger in the premenopausal women group (S2 Table). There was no significant difference in the prevalence of menopausal hormone therapy status between postmenopausal women with and without UL (S3 Table).

Table 2 shows the associations between dietary intake and the prevalence of UL in all participants, as analyzed through age-adjusted and two-stage multiple logistic regression models. Among all participants, higher fish intake showed an increased association with the prevalence of UL (Q4 vs. Q1: OR_2_ 1.68, 95% CI 1.01–2.81; *p* trend = 0.05). Higher poultry intake in Q2 and Q4 was associated with an increased prevalence of UL compared to Q1 (Q2 vs. Q1: OR_2_ 1.81, 95% CI 1.05–3.11; Q4 vs. Q1: OR_2_ 1.87, 95% CI 1.11–3.17), although the dose-response trend was not statistically significant (*p* for trend = 0.06).

**Table 2:** Odds ratios (ORs) and 95% confidence intervals (CIs) of the risk of uterine leiomyoma according to quartiles of intake of each food group in all participants.

Food intake	Median, g/d	Women with UL	Women without UL	Age-adjusted OR (95% CI)	*p* *	Multivariable adjusted OR_1_ (95% CI)	*p* *	Multivariable adjusted OR_2_ (95% CI)	*p* *
		220	452						
Vegetables and fruit									
Q1	73.10	56 (25.5)	112 (24.8)	1.00	0.69	1.00	0.72	1.00	0.77
Q2	189.00	61 (27.7)	107 (23.7)	1.14 (0.73–1.79)		1.16 (0.73–1.85)		1.15 (0.71–1.86)	
Q3	357.75	48 (21.8)	120 (26.6)	0.83 (0.52–1.32)		0.87 (0.53–1.43)		0.84 (0.50–1.40)	
Q4	728.12	55 (25.0)	113 (25.0)	0.98 (0.62–1.54)		0.99 (0.60–1.63)		0.99 (0.59–1.67)	
Vegetables									
Q1	36.96	50 (22.7)	118 (26.1)	1.00	0.96	1.00	0.86	1.00	0.64
Q2	82.57	57 (25.9)	111 (24.6)	1.22 (0.77–1.93)		1.26 (0.78–2.01)		1.39 (0.85–2.26)	
Q3	129.72	59 (26.8)	109 (24.1)	1.26 (0.8–2.00)		1.34 (0.83–2.18)		1.46 (0.89–2.41)	
Q4	239.64	54 (24.6)	114 (25.2)	1.06 (0.67–1.70)		1.11 (0.67–1.86)		1.23 (0.73–2.1)	
Fruit									
Q1	0	67 (30.5)	131 (29)	1.00	0.45	1.00	0.45	1.00	0.37
Q2	76.83	53 (24.1)	85 (18.8)	1.25 (0.8–1.97)		1.25 (0.79–1.99)		1.19 (0.74–1.92)	
Q3	230.57	47 (21.4)	121 (26.8)	0.79 (0.5–1.24)		0.82 (0.52–1.31)		0.78 (0.48–1.26)	
Q4	532.79	53 (24.1)	115 (25.4)	0.94 (0.6–1.46)		0.92 (0.58–1.45)		0.87 (0.54–1.40)	
Red meat									
Q1	5.83	59 (26.8)	109 (24.1)	1.00	0.33	1.00	0.26	1.00	0.17
Q2	18.33	55 (25.0)	118 (26.1)	0.90 (0.57–1.42)		0.92 (0.58–1.46)		0.93 (0.58–1.49)	
Q3	35.24	45 (20.5)	118 (26.1)	0.77 (0.48–1.25)		0.79 (0.48–1.30)		0.78 (0.47–1.30)	
Q4	78.13	61 (27.7)	107 (23.7)	1.17 (0.74–1.86)		1.25 (0.75–2.07)		1.34 (0.79–2.26)	
Processed meat									
T1	0	125 (56.8)	281 (62.2)	1.00	0.09	1.00	0.11	1.00	0.13
T2	0.67	27 (12.3)	42 (9.3)	1.58 (0.93–2.70)		1.64 (0.95–2.82)		1.47 (0.83–2.58)	
T3	3.33	68 (30.9)	129 (28.5)	1.46 (0.98–2.18)		1.46 (0.96–2.22)		1.43 (0.93–2.21)	
Poultry									
Q1	0	45 (20.5)	116 (25.7)	1.00	**0.05**	1.00	0.06	1.00	0.06
Q2	1.25	41 (18.6)	71 (15.7)	1.51 (0.90–2.53)		1.52 (0.90–2.56)		**1.81 (1.05–3.11)**	
Q3	2.50	66 (30.0)	146 (32.3)	1.30 (0.82–2.07)		1.33 (0.83–2.14)		1.50 (0.91–2.46)	
Q4	6.25	68 (30.9)	119 (26.3)	**1.72 (1.07–2.77)**		**1.71 (1.04–2.84)**		**1.87 (1.11–3.17)**	
Fish									
Q1	3.67	46 (20.9)	122 (27.0)	1.00	0.07	1.00	0.07	1.00	0.05
Q2	10.92	53 (24.1)	115 (25.4)	1.22 (0.76–1.95)		1.28 (0.79–2.07)		1.28 (0.77–2.11)	
Q3	19.55	58 (26.4)	110 (24.3)	1.41 (0.89–2.25)		1.49 (0.92–2.40)		1.55 (0.95–2.55)	
Q4	41.02	63 (28.6)	105 (23.2)	1.54 (0.97–2.45)		1.63 (1.00–2.67)		**1.68 (1.01–2.81)**	
Dairy product									
Q1	1.67	67 (30.5)	100 (22.1)	1.00	0.07	1.00	0.09	1.00	0.05
Q2	47.86	54 (24.6)	115 (25.4)	0.73 (0.46–1.14)		0.74 (0.47–1.17)		0.72 (0.45–1.16)	
Q3	120.00	49 (22.3)	119 (26.3)	**0.63 (0.40–0.99)**		**0.62 (0.39–0.99)**		**0.60 (0.37–0.98)**	
Q4	256.43	50 (22.7)	118 (26.1)	0.64 (0.40–1.00)		0.63 (0.39–1.03)		**0.58 (0.35–0.96)**	
Milk consumption									
Q1	0	76 (34.6)	123 (27.2)	1.00	0.17	1.00	0.19	1.00	0.08
Q2	16.67	44 (20.0)	76 (16.8)	0.97 (0.61–1.55)		0.95 (0.59–1.52)		0.95 (0.58–1.55)	
Q3	50.00	61 (27.7)	163 (36.1)	**0.62 (0.41–0.94)**		**0.61 (0.40–0.93)**		**0.57 (0.37–0.88)**	
Q4	200.00	39 (17.7)	90 (19.9)	0.71 (0.44–1.13)		0.70 (0.43–1.14)		0.61 (0.37–1.02)	
Ethanol		206	431						
T1	0	100 (48.8)	216 (50.0)	1.00	0.80	1.00	0.86	1.00	0.67
T2	0.87	41 (20.0)	68 (15.7)	1.38 (0.87–2.19)		1.34 (0.84–2.13)		1.38 (0.85–2.24)	
T3	8.16	64 (31.2)	148 (34.3)	1.03 (0.69–1.52)		1.03 (0.69–1.54)		1.18 (0.73–1.90)	

Q, quartile; T, tertile

Multivariable adjusted OR_1_was adjusted for age, BMI (kg/m^2^, <18.5, 18.5–23, 23–25, 25≤), total energy intake (kcal/d, quintile), and LDL-cholesterol (mg/dL, continuous)

Multivariable adjusted OR_2_ was adjusted for age, BMI (kg/m^2^, <18.5, 18.5–23, 23–25, 25≤), total energy intake (kcal/d, quintile), and LDL-cholesterol (mg/dL, continuous), early menarche (years old, ≤11, 11<), menopausal state (premenopausal, postmenopausal), parity and age at first delivery (nulliparity, years old, <25, 25≤), alcohol intake (g/d, continuous), smoking (never, ever), physical activity (MET-minute per week, tertile)

*p value for the test of trend of odds

Each of the cut-off values (g/d) were 121.9, 271.2, and 491.5 for vegetables and fruit intake; 60.8, 105.2, and 165.7 for vegetables intake; 0, 151.7, and 333.4 for fruit intake; 12.1, 25.0, and 47.5 for red meat intake; 0 and 0.7 for processed meat intake; 0, 1.3, and 3.5 for poultry intake; 7.1, 15.0, and 27.6 for fish intake; 22.1, 77.4, and 168.3 for dairy intake; 0, 33.4, and 100.0 for milk intake; and 0 and 1.95 for ethanol intake.

On the other hand, higher intake of dairy products in Q3 and Q4 exhibited a significant inverse association with the prevalence of UL compared to Q1 (Q3 vs. Q1: OR_2_ 0.60, 95% CI 0.37–0.98; Q4 vs. Q1: OR_2_ 0.58, 95% CI 0.35–0.96), but the dose-response relationship was not statistically significant (*p* trend = 0.05). The Q3 intake of milk showed a significant inverse association compared to Q1 (Q3 vs. Q1: OR_2_ 0.57, 95% CI 0.37–0.88).

**Fig 1:**
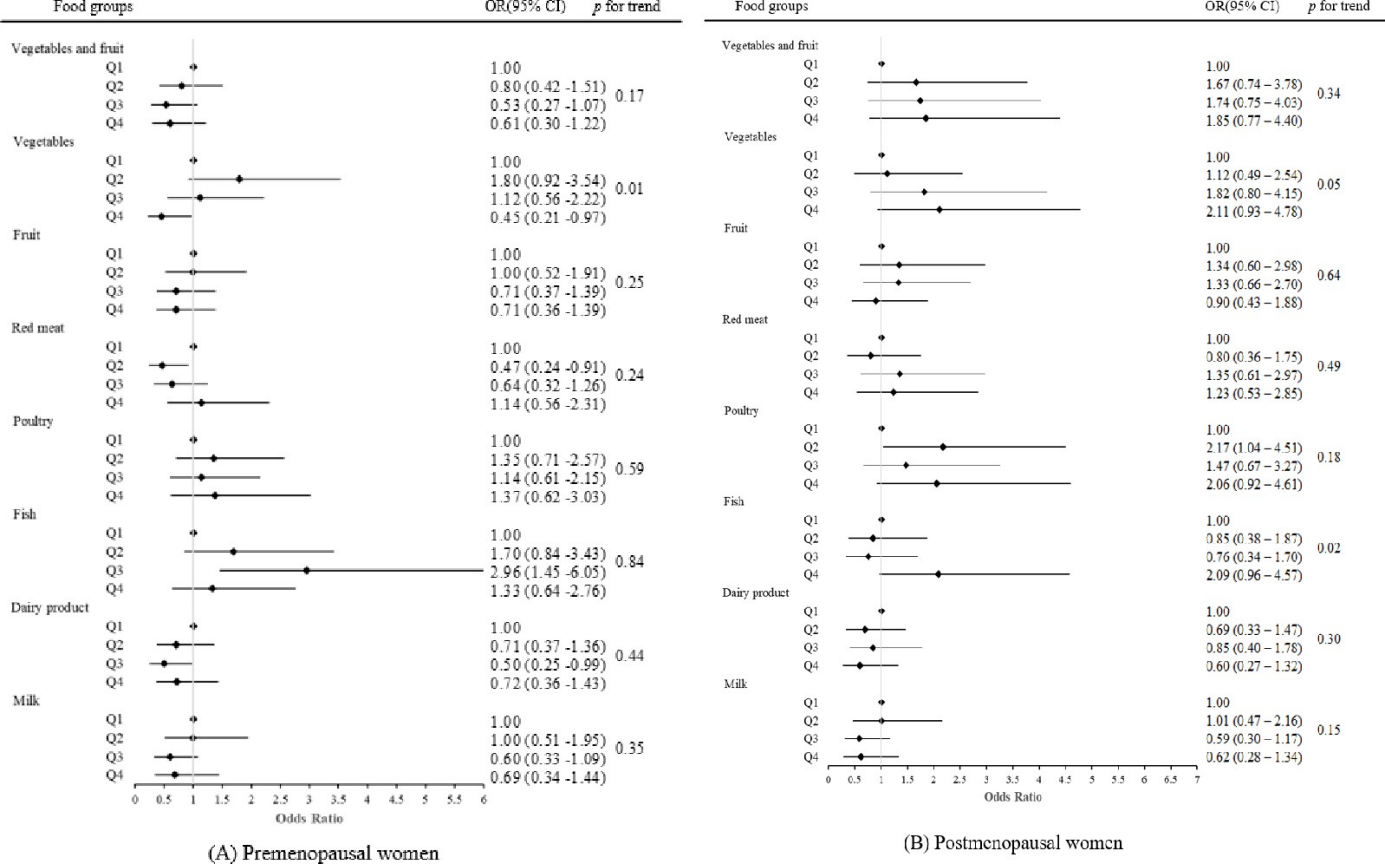
Association between dietary intake and the prevalence of UL in subgroups according to menopausal status.

A subgroup analysis was conducted for pre- and postmenopausal women (Table 3, Fig 1). In premenopausal women, vegetable intake was significantly inversely association with the UL prevalence in a dose-dependent manner (*p* trend = 0.01), with the highest quartile intake of vegetables showing statistical significance compared to the lowest quartile (Q4 vs. Q1; OR_2_ 0.45, 95% CI 0.21–0.97). Intake of red meat (Q2 vs. Q1: OR_2_ 0.47, 95% CI 0.24–0.91) and dairy products (Q3 vs. Q1: OR_2_ 0.50, 95% CI 0.25–0.99) showed an inverse association with UL prevalence. The intake of fish was significantly association with increased prevalence of UL (Q3 vs. Q1: OR_2_ 2.96, 95% CI 1.45–6.05).

**Table 3:** Odds ratios (ORs) and 95% confidence intervals (CIs) of the risk of uterine leiomyoma according to quartiles of intake of each food group in pre- and postmenopausal women.

	Premenopausal women						Postmenopausal women					
	Age-adjusted OR (95% CI)	*p* *	Multivariable adjusted OR_1_ (95% CI)	*p* *	Multivariable adjusted OR_2_ (95% CI)	*p* *	Age-adjusted OR (95% CI)	*p* *	Multivariable adjusted OR_1_ (95% CI)	*p* *	Multivariable adjusted OR_2_ (95% CI)	*p* *
Vegetables and fruit		**0.04**		0.14		0.17		0.10		0.26		0.34
Q1	1.00		1.00		1.00		1.00		1.00		1.00	
Q2	0.83 (0.45–1.52)		0.84 (0.45–1.57)		0.80 (0.42–1.51)		1.78 (0.84–3.78)		1.79 (0.81–3.95)		1.67 (0.74–3.78)	
Q3	**0.48 (0.25–0.91)**		0.54 (0.28–1.06)		0.53 (0.27–1.07)		1.73 (0.82–3.68)		1.77 (0.78–4.01)		1.74 (0.75–4.03)	
Q4	0.55 (0.29–1.02)		0.61 (0.31–1.19)		0.61 (0.30–1.22)		**2.14 (1.02–4.51)**		2.00 (0.85–4.66)		1.85 (0.77–4.40)	
Vegetables		**<0.01**		**0.01**		**0.01**		**<0.01**		**0.04**		0.05
Q1	1.00		1.00		1.00		1.00		1.00		1.00	
Q2	1.66 (0.89–3.09)		1.71 (0.90–3.28)		1.80 (0.92–3.54)		1.06 (0.49–2.32)		1.16 (0.52–2.61)		1.12 (0.49–2.54)	
Q3	0.94 (0.50–1.76)		1.04 (0.53–2.01)		1.12 (0.56–2.22)		1.79 (0.85–3.75)		1.93 (0.86–4.35)		1.82 (0.80–4.15)	
Q4	**0.47 (0.24–0.91)**		0.50 (0.24–1.05)		**0.45 (0.21–0.97)**		**2.34 (1.13–4.86)**		**2.23 (0.99–5.00)**		**2.11 (0.93–4.78)**	
Fruit		0.10		0.25		0.25		0.94		0.79		0.64
Q1	1.00		1.00		1.00		1.00		1.00		1.00	
Q2	1.12 (0.61–2.05)		1.05 (0.56–1.97)		1.00 (0.52–1.91)		1.39 (0.66–2.96)		1.36 (0.63–2.96)		1.34 (0.60–2.98)	
Q3	0.67 (0.36–1.25)		0.72 (0.38–1.38)		0.71 (0.37–1.39)		1.34 (0.69–2.60)		1.36 (0.68–2.70)		1.33 (0.66–2.70)	
Q 4	0.67 (0.36–1.23)		0.73 (0.38–1.40)		0.71 (0.36–1.39)		1.11 (0.56–2.19)		0.96 (0.47–1.98)		0.90 (0.43–1.88)	
Red meat		0.83		0.36		0.24		0.18		0.54		0.49
Q1	1.00		1.00		1.00		1.00		1.00		1.00	
Q2	0.55 (0.30–1.02)		**0.51 (0.27–0.95)**		**0.47 (0.24–0.91)**		0.88 (0.42–1.85)		0.82 (0.38–1.77)		0.8 (0.36–1.75)	
Q3	0.55 (0.29–1.04)		0.60 (0.31–1.17)		0.64 (0.32–1.26)		1.38 (0.67–2.86)		1.28 (0.59–2.77)		1.35 (0.61–2.97)	
Q4	0.87 (0.46–1.61)		1.07 (0.54–2.09)		1.14 (0.56–2.31)		1.49 (0.73–3.07)		1.2 (0.53–2.7)		1.23 (0.53–2.85)	
Processed meat		0.22		0.19		0.18						
T1	1.00		1.00		1.00		1.00		1.00		1.00	
T2	1.04 (0.57–1.91)		1.16 (0.62–2.17)		1.26 (0.66–2.42)		**2.33 (1.28–4.25)**		**2.12 (1.13–3.95)**		**2.33 (1.21–4.49)**	
T3	1.37 (0.81–2.32)		1.46 (0.84–2.54)		1.51 (0.85–2.68)							
Poultry		0.96		0.64		0.59		**0.04**		0.15		0.18
Q1	1.00		1.00		1.00		1.00		1.00		1.00	
Q2	1.14 (0.63–2.09)		1.24 (0.67–2.31)		1.35 (0.71–2.57)		**2.18 (1.08–4.41)**		**2.16 (1.06–4.42)**		**2.17 (1.04–4.51)**	
Q3	1.02 (0.58–1.81)		1.11 (0.60–2.03)		1.14 (0.61–2.15)		1.45 (0.68–3.08)		1.40 (0.65–3.04)		1.47 (0.67–3.27)	
Q4	1.07 (0.53–2.17)		1.29 (0.60–2.77)		1.37 (0.62–3.03)		**2.48 (1.21–5.09)**		2.08 (0.96–4.50)		2.06 (0.92–4.61)	
Fish		0.68		0.86		0.84		**<0.01**		**0.02**		**0.02**
Q1	1.00		1.00		1.00		1.00		1.00		1.00	
Q2	1.19 (0.63–2.27)		1.50 (0.76–2.95)		1.70 (0.84–3.43)		0.93 (0.44–1.96)		0.90 (0.42–1.95)		0.85 (0.38–1.87)	
Q3	**2.04 (1.08–3.84)**		**2.78 (1.40–5.53)**		**2.96 (1.45–6.05)**		0.82 (0.38–1.76)		0.82 (0.37–1.81)		0.76 (0.34–1.70)	
Q4	0.92 (0.48–1.79)		1.25 (0.62–2.53)		1.33 (0.64–2.76)		**2.35 (1.17–4.73)**		2.11 (0.98–4.54)		2.09 (0.96–4.57)	
Dairy product		0.15		0.46		0.44		0.86		0.45		0.30
Q1	1.00		1.00		1.00		1.00		1.00		1.00	
Q2	0.69 (0.38–1.27)		0.74 (0.40–1.37)		0.71 (0.37–1.36)		0.71 (0.34–1.45)		0.68 (0.32–1.43)		0.69 (0.33–1.47)	
Q3	0.56 (0.30–1.05)		0.60 (0.31–1.16)		**0.50 (0.25–0.99)**		0.93 (0.47–1.88)		0.85 (0.41–1.77)		0.85 (0.40–1.78)	
Q4	0.60 (0.32–1.11)		0.74 (0.38–1.43)		0.72 (0.36–1.43)		0.83 (0.41–1.68)		0.67 (0.31–1.42)		0.60 (0.27–1.32)	
Milk		0.13		0.36		0.35		0.54		0.28		0.15
Q 1	1.00		1.00		1.00		1.00		1.00		1.00	
Q2	0.96 (0.51–1.83)		0.98 (0.51–1.88)		1.00 (0.51–1.95)		0.99 (0.47–2.07)		1.04 (0.49–2.20)		1.01 (0.47–2.16)	
Q3	0.61 (0.35–1.07)		0.64 (0.36–1.15)		0.60 (0.33–1.09)		0.67 (0.35–1.28)		0.62 (0.32–1.21)		0.59 (0.30–1.17)	
Q4	0.60 (0.31–1.17)		0.71 (0.35–1.44)		0.69 (0.34–1.44)		0.86 (0.42–1.74)		0.72 (0.34–1.51)		0.62 (0.28–1.34)	
Alcohol		0.79		0.61		0.42		0.74		0.79		0.95
T1	1.00		1.00		1.00		1.00		1.00		1.00	
T2	1.53 (0.87–2.69)		1.46 (0.82–2.60)		1.67 (0.91–3.08)		1.60 (0.54–4.78)		1.91 (0.61–5.95)		2.15 (0.68–6.83)	
T3	1.22 (0.71–2.10)		1.28 (0.73–2.25)		1.54 (0.74–3.23)		0.94 (0.53–1.67)		0.97 (0.54–1.75)		1.03 (0.53–2.01)	

Q, quartile; T, tertile

Multivariable adjusted OR_1_was adjusted for age, BMI (kg/m^2^, <18.5, 18.5–23, 23–25, 25≤), total energy intake (kcal/d, quintile), and LDL-cholesterol (mg/dL, continuous)

Multivariable adjusted OR_2_ was adjusted for age, BMI (kg/m^2^, <18.5, 18.5–23, 23–25, 25≤), total energy intake (kcal/d, quintile), and LDL-cholesterol (mg/dL, continuous), early menarche (years old, ≤11, 11<), parity and age at first delivery (nulliparity, years old, <25, 25≤), alcohol intake (g/d, continuous), smoking (never, ever), physical activity (MET-minute per week, tertile)

*p value for the test of trend of odds

Each of the cut-off values (g/d) among premenopausal women were 123.8, 281.2, and 474.2 for vegetables and fruit intake; 55.2, 91.5, and 153.4 for vegetables intake; 0, 171.4, and 356.5 for fruit intake; 14.2, 31.3, and 55.7 for red meat intake; 0 and 1.4 for processed meat intake; 1.3, 2.6, and 6.3 for poultry intake; 6.9, 14.1, and 25.4 for fish intake; 25.5, 72.9, and 161.7 for dairy intake; 0, 33.4, and 100.1 for milk intake; and 0 and 3.8 for ethanol intake.

Each of the cut-off values (g/d) among postmenopausal women were 118.7, 255.5, and 520.4 for vegetables and fruit intake; 67.6, 118.0, and 191.1 for vegetables intake; 0, 120.0, and 304.0 for fruit intake; 8.4, 18.8, and 37.0 for red meat intake; 0 for processed meat intake; 0, 1.26, and 3.2 for poultry intake; 7.3, 16.3, and 29.7 for fish intake; 20.1, 85.0, and 175.4 for dairy intake; 0, 21.5, and 100.1 for milk intake; and 0 and 0.8 for ethanol intake.

In postmenopausal women, higher fish intake was significantly associated with higher UL prevalence (*p* trend = 0.02), although no statistically significant association was demonstrated in each quartile group. Intake of processed meat (top vs. bottom: OR_2_ 2.33, 95% CI 1.21–4.49) and poultry (Q2 vs. Q1: OR_2_ 2.17, 95% CI 1.04–4.51) showed a significant association with an increased UL prevalence.

## Discussion

When summarizing our study findings comprehensively, elevated fish and poultry consumption were associated with a higher prevalence of UL, with odds ratios (95% confidence intervals) comparing the top vs. bottom quartiles of 1.68 (1.01–2.81; *p* trend = 0.05) for fish intake and 1.87 (1.11–3.17; *p* trend = 0.06) for poultry intake. Conversely, a higher intake of dairy products displayed an inverse association with UL prevalence (OR 0.58, 95% CI 0.35–0.96; *p* trend = 0.05). Upon analyzing pre- and post-menopausal women separately, a similar pattern emerged with increased prevalence associated with fish intake and decreased prevalence linked to dairy product intake. However, the association between poultry intake and UL prevalence was mainly evident among postmenopausal women. Among premenopausal women, a higher vegetable intake was associated with a lower prevalence of UL (OR 0.45, 95% CI 0.21–0.97 for top vs. bottom quartiles; *p* trend = 0.01).

We observed a significant association between fish consumption and UL in premenopausal women (Q3 vs. Q1: OR2 2.96, 95% CI 1.45–6.05) and a dose-dependent association in postmenopausal women (Q4 vs. Q1: OR2 2.09, 95% CI 0.96–4.57; *p* trend = 0.02). Contrasting findings exist in the literature, with an Italian case-control study reporting an inverse association [24], while Chinese and Japanese studies found no significant differences [25, 26]. A US cohort study demonstrated a 1.2-fold increased prevalence of UL in women who consumed sport fish from the Great Lakes for a decade, suggesting a potential risk elevation due to polychlorinated biphenyls (PCBs) exposure from fish consumption [27]. However, a recent prospective cohort study investigating the relationship between PCBs and UL found no significant correlation [28]. Most previous studies have predominantly analyzed fish consumption in terms of dietary fat [15]. For instance, a prospective study involving 1,171 premenopausal African-American women in the US indicated that intakes of total fat, saturated fat, monounsaturated fat, polyunsaturated fat, and trans-fat were not appreciably associated with UL incidence. Interestingly, the consumption of marine ω-3 polyunsaturated fatty acid, specifically docosahexaenoic acid, was linked to a 49% higher UL incidence (Q4 vs. Q1: HR 1.49, 95% CI 1.04, 2.14, *p* trend = 0.01) [29]. Nevertheless, based solely on the results of this study, it is challenging to estimate the nutritional components of fish contributing to the association between fish consumption and UL. This point applies to all the associations between food groups included in this study and UL, and it has been reiterated as a limitation of this research.

Vegetable intake demonstrated a significant protective association with the prevalence of UL in premenopausal women. The odds ratio of the highest quartile of vegetable intake compared to the bottom quartile was 0.45 (95% CI 0.21–0.97), with a significant dose-dependent relationship (*p* trend = 0.01). These findings align with previous research, such as the Black Women’s Health Study, indicating reduced risk of UL development with higher fruit and vegetable consumption (four or more servings of fruits or vegetables daily, IRR = 0.90, 95% CI 0.82–0.98) [30]. In a case-control study involving 273 women, of whom 94% were of Han Chinese ethnicity, a negative correlation was found between vegetable and fruit intake and UL (OR 0.5, 95% CI 0.3–0.9) in premenopausal women [25]. Furthermore, other investigations have indicated that women with UL consume green vegetables and fruits less frequently than women without UL [24, 31]. These protective associations are attributed to mechanisms such as decreased bioavailable estrogen and growth factors [32, 33], or elevated levels of phytochemicals with anti-inflammatory properties [34, 35]. However, this study did not observe a protective effect of fruit consumption. The odds ratios for combined vegetable and fruit intake, as well as fruit intake alone, were 0.61 (95% CI 0.30–1.22) and 0.71 (95% CI 0.36–1.39) for the highest quartile over the lowest quartile, respectively. This finding aligns with a case-control study involving 843 Italian women, which indicated that vegetables were more protective than fruits against UL prevalence (OR 0.5, 95% CI 0.4–0.6 for green vegetables, OR 0.8, 95% CI 0.6–1.0 for fruit consumption) [24].

We observed a protective association between dairy consumption and UL prevalence (Q4 vs. Q1: OR_2_ 0.58, 95% CI 0.35–0.96 for all participants; Q3 vs. Q1: OR_2_ 0.50, 95% CI 0.25–0.99 for premenopausal women). These findings align with a previous study that reported a protective effect of frequent consumption of milk and low-fat dairy products, as well as a modest protective effect for yogurt consumption, against the occurrence of UL. However, no significant associations were found for butter, cheese, and ice cream among African American women [36]. On the contrary, an Italian study presented contrasting results, finding no association between milk and cheese intake and the risk of UL [24]. Additionally, a Chinese prospective cohort study demonstrated an increased risk when analyzing combined milk and soymilk consumption [37]. Dairy products are complex compounds with composition variations influenced by regional disparities in livestock production environments. Furthermore, reports suggest that dairy products might contain estrogenic compounds that could be absorbed and affect the menstrual cycle [38, 39]. As a result, diverse research findings have emerged concerning the link between dairy consumption and the risk of UL. In a substantial prospective cohort study spanning 18 years, no distinct associations emerged between overall dairy consumption and the risk of UL. Nevertheless, the study did establish that yogurt intake and dietary calcium were associated with a reduced risk of UL development [40].

Regarding the connection between meat consumption and UL, we observed a protective association with certain levels of red meat intake in premenopausal women (Q2 vs. Q1: OR_2_ 0.47, 95% CI 0.24–0.91). On the other hand, we identified an increased association between processed meat (higher vs. lower: OR_2_ 2.33, 95% CI 1.21–4.49) and poultry (Q2 vs. Q1: OR_2_ 2.17, 95% CI 1.04–4.51) consumption and UL prevalence in postmenopausal women. This aligns with findings from an Italian case-control study, which demonstrated that significant consumption of meats such as beef or ham was associated with an elevated risk of UL [24]. However, this risk was found to be insignificant in the Chinese population [25]. To classify meats, we categorized them into red meat, processed meat, and poultry. Processed meat and poultry intake exhibited an association with increased UL prevalence exclusively in postmenopausal women, whereas red meat intake indicated a lower UL prevalence solely in premenopausal women. Interpreting these associations is limited by the relatively low absolute amount of meat intake within this population. Nonetheless, it appears that processed meat, rather than red meat, may contain specific metabolites that could stimulate proliferative activities in UL cells [41, 42].

A notable finding in our study was the variation in the association between dietary patterns and UL prevalence based on menopausal status. Numerous studies have reported differing dietary impacts on hormone-related conditions contingent on menopausal status. For instance, an investigation into the impact of a diabetes risk reduction diet on endometrial cancer revealed inverse associations exclusively among postmenopausal women, without such effects seen in premenopausal women [43]. Similarly, a study exploring the link between urinary isoflavone and urinary estrogen levels after isoflavone intake identified a positive correlation solely in postmenopausal women. Cumulative evidence from epidemiological and metabolomics research suggests that the postmenopausal state can influence a specific set of metabolites in response to a particular diet, distinct from the premenopausal state [44, 45]. The potential anti-proliferative effect of a diet might be more profound in the high estrogenic environment of premenopausal women. Conversely, the impact of certain dietary components, such as estrogenic compounds in fatty fish or processed meat, could be more significant in the hypoestrogenic context of postmenopausal women. Consequently, considering the influence of menopausal status is imperative when investigating the relationship between dietary intake and health outcomes.

To the best of our knowledge, this is the first Korean study to investigate the association between dietary factors and UL, employing a validated Food Frequency Questionnaire (FFQ) known for its commendable reproducibility and validity [20]. All UL cases and non-cases underwent diagnosis through pelvic ultrasound examination, considered the most sensitive and clinically effective diagnostic tool for UL [46]. Notably, the assessment of dietary intake data coincided with clinical factors, with the analysis accounting for menopausal status and other relevant confounding factors.

However, several limitations must be acknowledged. First, despite categorizing a substantial number of participants as patients, including those with at least one UL measuring 10 mm or larger, this cannot be considered a clinically significant classification based on associated symptoms or factors such as their number and size, as well as information about previous excision surgeries. Additionally, data on hormonal therapies, such as oral contraceptive use, which could impact past UL size changes, were missing. Furthermore, this study excluded women who had undergone hysterectomy, inadvertently excluding severe or symptomatic cases.

Secondly, regarding the study design and population, inevitable information discrepancies may arise from data gathered through self-reported food intake questionnaires. The cross-sectional nature of the study limits the ability to infer causal relationships, raising the possibility that those already diagnosed with ULs may exhibit specific dietary patterns. Furthermore, the study may be subject to potential bias toward individuals of medium to high socioeconomic status who willingly invested USD $500–1300 for private health assessments, influencing the findings due to their increased health awareness and motivation towards adopting healthier lifestyles. Additionally, the single-center design warrants caution in generalizing these findings to the entirety of Korean women.

Thirdly, while the research analyzing the association between dietary patterns and the occurrence of diseases is meaningful in identifying correlations at a broader level [47], our study specifically focused on food groups and did not thoroughly examine the impact of nutrients derived from foods on dietary intake analyses. Notably, data regarding the concentration of associated metabolites, blood markers of inflammation, and reproductive or growth hormones—presumed to play a mediating role in these associations—were lacking. Recognizing this as the primary limitation of our current study, it is imperative to consider avenues for future research that explores individual nutrients within these groups, potentially providing valuable insights. Based on our findings, we are particularly interested in further investigating the associations of protein, calcium, and fiber intakes with ULs.

## Conclusions

In our study involving Korean women who underwent pelvic ultrasonography, we found that high consumption of fish and poultry, coupled with low intake of dairy products, correlated with an elevated prevalence of UL. Furthermore, vegetable intake exhibited an inverse relationship with UL prevalence, particularly among premenopausal women. These results suggest that dietary interventions offer promise as a potential preventive strategy for UL, with a specific focus on premenopausal women who are disproportionately affected by this prevalent and consequential gynecological condition.

## Supporting information

S1 Table Median values of the tertiles or quartiles of each dietary group in all, pre-, and postmenopausal women. (DOCX)

S2 Table The distribution of size and number of uterine leiomyomas classified by menopausal status. (DOCX)

S3 Table The distribution of hormone therapy according to the presence or absence of uterine leiomyomas in postmenopausal women. (DOCX)
