# Supplementary material for: Dietary intake is associated with the prevalence of uterine leiomyoma in Korean women: A retrospective cohort study
Source: PLoS One. 2024 Feb 15;19(2):e0291157. doi: 10.1371/journal.pone.0291157 (PMC10868850; doi:10.1371/journal.pone.0291157)
Supplement: S1 Table — (DOCX) [file pone.0291157.s001.docx]

**S1 Table.** **Median values of the tertiles or quartiles of each dietary group in all, pre-, and postmenopausal women.**

|  | All Participants, n (%) | | |  | Premenopausal Participants, n (%) | | |  | Postmenopausal participants, n (%) | | |
| --- | --- | --- | --- | --- | --- | --- | --- | --- | --- | --- | --- |
| Food group | Amount | Women with UL | Women without UL |  | Amount | Women with UL | Women without UL |  | Amount | Women with UL | Women without UL |
| Vegetables and fruit | Medians, g/d | 220 | 452 |  | Medians, g/d | 133 | 250 |  | Medians, g/d | 87 | 202 |
| Q1 | 73.10 | 56 (25.5) | 112 (24.8) |  | 66.96 | 41 (30.8) | 54 (21.6) |  | 89.56 | 15 (17.2) | 57 (28.2) |
| Q2 | 189.00 | 61 (27.7) | 107 (23.7) |  | 190.88 | 37 (27.8) | 59 (23.6) |  | 185.47 | 23 (26.4) | 49 (24.3) |
| Q3 | 357.75 | 48 (21.8) | 120 (26.6) |  | 363.15 | 26 (19.6) | 70 (28) |  | 339.78 | 23 (26.4) | 50 (24.8) |
| Q4 | 728.12 | 55 (25.0) | 113 (25.0) |  | 736.18 | 29 (21.8) | 67 (26.8) |  | 721.91 | 26 (29.9) | 46 (22.8) |
| Vegetables |  |  |  |  |  |  |  |  |  |  |  |
| Q1 | 36.96 | 50 (22.7) | 118 (26.1) |  | 35.5 | 34 (25.6) | 61 (24.4) |  | 42.96 | 16 (18.4) | 56 (27.7) |
| Q2 | 82.57 | 57 (25.9) | 111 (24.6) |  | 73.13 | 41 (30.8) | 55 (22) |  | 94.51 | 17 (19.5) | 55 (27.2) |
| Q3 | 129.72 | 59 (26.8) | 109 (24.1) |  | 119.39 | 34 (25.6) | 62 (24.8) |  | 142.86 | 25 (28.7) | 48 (23.8) |
| Q4 | 239.64 | 54 (24.6) | 114 (25.2) |  | 211.85 | 24 (18.1) | 72 (28.8) |  | 265.46 | 29 (33.3) | 43 (21.3) |
| Fruit |  |  |  |  |  |  |  |  |  |  |  |
| Q1 | 0 | 67 (30.5) | 131 (29) |  | 0 | 41 (30.8) | 60 (24) |  | 0 | 26 (29.9) | 71 (35.2) |
| Q2 | 76.83 | 53 (24.1) | 85 (18.8) |  | 92.27 | 36 (27.1) | 54 (21.6) |  | 51.67 | 16 (18.4) | 31 (15.4) |
| Q3 | 230.57 | 47 (21.4) | 121 (26.8) |  | 243.68 | 28 (21.1) | 68 (27.2) |  | 212.84 | 24 (27.6) | 49 (24.3) |
| Q4 | 532.79 | 53 (24.1) | 115 (25.4) |  | 211.88 | 28 (21.1) | 68 (27.2) |  | 504.60 | 21 (24.1) | 51 (25.3) |
| Red meat |  |  |  |  |  |  |  |  |  |  | 19 (21.8) |
| Q1 | 5.83 | 59 (26.8) | 109 (24.1) |  | 8.33 | 43 (32.3) | 48 (19.2) |  | 2.50 | 19 (21.8) |  |
| Q2 | 18.33 | 55 (25.0) | 118 (26.1) |  | 22.50 | 30 (22.6) | 73 (29.2) |  | 14.38 | 18 (20.7) | 56 (27.7) |
| Q3 | 35.24 | 45 (20.5) | 118 (26.1) |  | 40.42 | 26 (19.6) | 67 (26.8) |  | 25.00 | 24 (27.6) | 47 (23.3) |
| Q4 | 78.13 | 61 (27.7) | 107 (23.7) |  | 86.55 | 34 (25.6) | 62 (24.8) |  | 66.73 | 26 (29.9) | 47 (23.3) |
| Processed meat |  |  |  |  |  |  |  |  |  |  |  |
| T1 | 0 | 125 (56.8) | 281 (62.2) |  | 0 | 64 (48.1) | 110 (44) |  | 0 | 61 (70.1) | 171 (84.7) |
| T2 | 0.67 | 27 (12.3) | 42 (9.3) |  | 0.67 | 25 (18.8) | 47 (18.8) |  | 1.67 | 26 (29.9) | 31 (15.3) |
| T3 | 3.33 | 68 (30.9) | 129 (28.5) |  | 3.33 | 44 (33.1) | 93 (37.2) |  |  |  |  |
| Poultry |  |  |  |  |  |  |  |  |  |  |  |
| Q1 | 0 | 45 (20.5) | 116 (25.7) |  | 0 | 42 (31.6) | 70 (28) |  | 0 | 19 (21.8) | 73 (36.1) |
| Q2 | 1.25 | 41 (18.6) | 71 (15.7) |  | 2.50 | 33 (24.8) | 59 (23.6) |  | 1.25 | 25 (28.7) | 44 (21.8) |
| Q3 | 2.50 | 66 (30.0) | 146 (32.3) |  | 6.25 | 39 (29.3) | 76 (30.4) |  | 2.50 | 17 (19.5) | 45 (22.3) |
| Q4 | 6.25 | 68 (30.9) | 119 (26.3) |  | 8.54 | 19 (14.3) | 45 (18) |  | 6.25 | 26 (29.9) | 40 (19.8) |
| Fish |  |  |  |  |  |  |  |  |  |  |  |
| Q1 | 3.67 | 46 (20.9) | 122 (27.0) |  | 3.33 | 26 (19.6) | 69 (27.6) |  | 4.38 | 19 (21.8) | 53 (26.2) |
| Q2 | 10.92 | 53 (24.1) | 115 (25.4) |  | 9.90 | 35 (26.3) | 62 (24.8) |  | 11.89 | 18 (20.7) | 54 (26.7) |
| Q3 | 19.55 | 58 (26.4) | 110 (24.3) |  | 18.07 | 41 (30.8) | 54 (21.6) |  | 21.52 | 17 (19.5) | 56 (27.7) |
| Q4 | 41.02 | 63 (28.6) | 105 (23.2) |  | 38.45 | 31 (23.3) | 65 (26) |  | 45.45 | 33 (37.9) | 39 (19.3) |
| Dairy product |  |  |  |  |  |  |  |  |  |  |  |
| Q1 | 1.67 | 67 (30.5) | 100 (22.1) |  | 7.33 | 43 (32.3) | 52 (20.8) |  | 0 | 24 (27.6) | 48 (23.8) |
| Q2 | 47.86 | 54 (24.6) | 115 (25.4) |  | 51.00 | 33 (24.8) | 63 (25.2) |  | 46.17 | 19 (21.8) | 53 (26.2) |
| Q3 | 120.00 | 49 (22.3) | 119 (26.3) |  | 114.00 | 26 (19.6) | 70 (28) |  | 125.71 | 23 (26.4) | 50 (24.8) |
| Q4 | 256.43 | 50 (22.7) | 118 (26.1) |  | 258.02 | 31 (23.3) | 65 (26) |  | 255.12 | 21 (24.1) | 51 (25.3) |
| Milk |  |  |  |  |  |  |  |  |  |  |  |
| Q1 | 0 | 76 (34.6) | 123 (27.2) |  | 0 | 44 (33.1) | 59 (23.6) |  | 0 | 32 (36.8) | 64 (31.7) |
| Q2 | 16.67 | 44 (20.0) | 76 (16.8) |  | 16.67 | 28 (21.1) | 43 (17.2) |  | 16.67 | 16 (18.4) | 32 (15.8) |
| Q3 | 50.00 | 61 (27.7) | 163 (36.1) |  | 50.00 | 39 (29.3) | 98 (39.2) |  | 82.14 | 22 (25.3) | 66 (32.7) |
| Q4 | 200.00 | 39 (17.7) | 90 (19.9) |  | 200.00 | 22 (16.5) | 50 (20) |  | 200.00 | 17 (19.5) | 40 (19.8) |
| Alcohol |  | 206 | 431 |  |  | 242 | 217 |  |  | 78 | 190 |
| T1 | 0 | 100 (48.8) | 216 (50.0) |  | 0 | 53 (41.7) | 100 (41.3) |  | 0 | 47 (60.3) | 116 (61.1) |
| T2 | 0.87 | 41 (20.0) | 68 (15.7) |  | 1.37 | 36 (28.4) | 57 (23.6) |  | 0.32 | 6 (7.7) | 9 (4.7) |
| T3 | 8.16 | 64 (31.2) | 148 (34.3) |  | 12.15 | 38 (29.9) | 85 (35.1) |  | 3.79 | 25 (32.1) | 65 (34.2) |
