# Supplementary material for: Dietary intake is associated with the prevalence of uterine leiomyoma in Korean women: A retrospective cohort study
Source: PLoS One. 2024 Feb 15;19(2):e0291157. doi: 10.1371/journal.pone.0291157 (PMC10868850; doi:10.1371/journal.pone.0291157)
Supplement: S2 Table — (DOCX) [file pone.0291157.s002.docx]

S2 Table. The distribution of size and number of uterine leiomyomas classified by menopausal status.

|  | Women with UL, premenopause  (n=133) | Women with UL, postmenopause  (n=87) | *p*-value |
| --- | --- | --- | --- |
| Largest diameter of leiomyoma, cm | 2.6±1.5 | 2.0±1.0 | <0.001 |
| Mean no. of leiomyoma | 1.8±1.2 | 1.7±1.0 | 0.305 |
| Women with single nodule, n (%) | 76 (57.1) | 54 (62.1) | 0.486 |
| Women with two or more nodules, n (%) | 57(42.9) | 33 (37.9) |  |
