# Supplementary material for: Dietary intake is associated with the prevalence of uterine leiomyoma in Korean women: A retrospective cohort study
Source: PLoS One. 2024 Feb 15;19(2):e0291157. doi: 10.1371/journal.pone.0291157 (PMC10868850; doi:10.1371/journal.pone.0291157)
Supplement: S3 Table — (DOCX) [file pone.0291157.s003.docx]

S3 Table. The distribution of hormone therapy according to the presence or absence of uterine leiomyomas in postmenopausal women.

|  | Postmenopausal women without UL  (n=202) | Postmenopausal women with UL  (n=87) | *p*-value |
| --- | --- | --- | --- |
| Menopausal hormone therapy |  |  | 0.377 |
| Never-user | 161 (79.7) | 66 (75.9) |  |
| Current-user | 30 (14.9) | 13 (14.9) |  |
| Past-user | 11(5.4) | 7 (8.0) |  |
